# Asymmetric Dimethylarginine as a Potential Mediator in the Association between Periodontitis and Cardiovascular Disease: A Systematic Review of Current Evidence

**DOI:** 10.3390/dj12090297

**Published:** 2024-09-21

**Authors:** Biagio Rapone, Francesco Inchingolo, Giulia Margherita Tartaglia, Maurizio De Francesco, Elisabetta Ferrara

**Affiliations:** 1 Interdisciplinary Department of Medicine, University of Bari, 70121 Bari, Italy; francesco.inchingolo@uniba.it; 2 School of Medicine, European University of Madrid, 28670 Madrid, Spain; giugitartaglia@gmail.com; 3 Department of Neurosciences, Institute of Clinical Dentistry, University of Padua, 35128 Padua, Italy; maurizio.defrancesco@unipd.it; 4 Department of Human Sciences, Law, and Economics, Telematic University Leonardo da Vinci, UNIDAV, Torrevecchia Teatina, 66100 Chieti, Italy; elisabetta.ferrara@unidav.it

**Keywords:** asymmetric dimethylarginine (ADMA), cardiovascular disease, endothelial dysfunction, inflammation, nitric oxide synthase, oxidative stress, periodontitis

## Abstract

**Background:** Periodontitis, a chronic inflammatory disease, has been associated with an elevated risk of cardiovascular disease (CVD). Asymmetric dimethylarginine (ADMA), an endogenous inhibitor of nitric oxide synthase, has emerged as a potential biomarker linking periodontitis, endothelial dysfunction, and CVD. This systematic review aimed to synthesize the existing evidence on the relationship between ADMA, periodontitis, and CVD, and to evaluate ADMA’s potential as a biomarker for periodontal disease progression and its correlation with endothelial dysfunction. **Methods:** A comprehensive literature search was conducted in PubMed, Scopus, and Web of Science databases from their inception to March 2023. Observational and interventional studies assessing ADMA levels in patients with periodontitis and/or CVD were included. The methodological quality of the included studies was evaluated using the NIH Quality Assessment Tools. Due to the heterogeneity of the included studies, a qualitative synthesis was performed. **Results:** Cross-sectional studies consistently demonstrated significantly elevated ADMA levels in patients with periodontitis and CVD compared to healthy controls. The prospective cohort study indicated that successful periodontal treatment was associated with a significant reduction in ADMA levels and concomitant improvement in endothelial function. The pilot cohort study reported a significant decrease in ADMA levels following periodontal therapy in patients with chronic kidney disease. However, the randomized controlled trials did not demonstrate significant alterations in ADMA levels or endothelial function subsequent to periodontal treatment in patients with periodontitis alone. **Conclusions**: Periodontal treatment may effectively reduce ADMA levels and improve endothelial function, particularly in patients with comorbidities. These findings suggest that ADMA is a promising biomarker linking periodontitis, endothelial dysfunction, and CVD. However, the limitations of this study include the small number of studies, heterogeneity in the study designs, and a lack of long-term follow-up data. Further high-quality, longitudinal studies are required to confirm its clinical utility and elucidate the underlying mechanisms of these relationships. The integration of periodontal care into CVD prevention and management strategies warrants consideration, as it may contribute to mitigating the cardiovascular risk associated with periodontitis.

## 1. Introduction

Periodontitis, a chronic inflammatory disease caused by periodontal bacteria, is a significant global health concern affecting approximately 50% of adults in the USA aged over 30 years, with nearly 10% of the world population suffering from a severe form of the disease [1,2]. In recent years, the link between periodontitis and cardiovascular health has gained increasing recognition [3,4].

Observational studies have demonstrated a clear association between periodontitis and coronary heart disease (CHD), including myocardial infarction, stroke, and peripheral vascular disease [5,6,7]. Large cohort studies and systematic reviews have highlighted a graded association between periodontitis, tooth loss, and an increased risk of CHD and stroke [8].

The chronic inflammatory condition associated with periodontitis may play a role in the initiation and progression of atherosclerosis and cardiovascular diseases (CVDs) through various pathways [9]. These pathways encompass endothelial dysfunction, oxidative stress, and systemic inflammation. A key player in this relationship is nitric oxide (NO) [10]. This signaling molecule is crucial for maintaining endothelial function and vascular homeostasis. Endothelial nitric oxide synthase (eNOS) synthesizes NO from L-arginine [11]. Once produced, NO exerts multiple beneficial effects on the cardiovascular system: it functions as a potent vasodilator, exhibits anti-inflammatory properties, inhibits platelet aggregation, and counteracts smooth muscle cell proliferation [12,13,14,15,16]. Asymmetric dimethylarginine (ADMA), an endogenous inhibitor of NOS, can lead to endothelial dysfunction when elevated [17]. ADMA is formed by the methylation of arginine residues in proteins by protein arginine methyltransferases (PRMTs) and is degraded by the enzyme dimethylarginine dimethylaminohydrolase (DDAH) [13]. An imbalance between ADMA production and degradation can result in increased ADMA levels, contributing to various pathological conditions associated with endothelial dysfunction [18,19,20].

Recent evidence suggests that ADMA may play a role in the link between periodontitis and CVD. Studies have shown that patients with periodontitis have higher levels of ADMA compared to healthy controls [15,16,21,22,23]. The chronic inflammatory state in periodontitis is thought to contribute to increased ADMA levels through several potential mechanisms, including increased PRMT activity, decreased DDAH activity, and increased oxidative stress [24,25].

While previous studies have focused on serum ADMA levels, there is a lack of research evaluating salivary ADMA levels during periodontitis. Moreover, there is insufficient data on the impact of periodontitis on serum ADMA levels in patients with both periodontitis and CVD [26,27,28,29,30].

The objectives of this systematic review were threefold: (1) to investigate the association between ADMA levels and the presence of periodontitis and CVD; (2) to evaluate the potential of ADMA as a biomarker for periodontal disease progression and its association with endothelial dysfunction; and (3) to assess the impact of periodontal treatment on ADMA levels and endothelial function in patients with periodontitis and/or CVD.

## 2. Materials and Methods

### 2.1. Search Strategy and Selection Criteria

This systematic review was conducted in accordance with the Preferred Reporting Items for Systematic Reviews and Meta-Analyses (PRISMA) guidelines [31]. A comprehensive literature search was performed using PubMed, Scopus, and Web of Science databases from inception to March 2023. The search strategy included a combination of keywords and Medical Subject Headings (MeSH) terms related to periodontitis, cardiovascular disease, asymmetric dimethylarginine, and endothelial dysfunction. The full electronic search syntax for the PubMed database was the following: ((“periodontal diseases”[MeSH Terms] OR (“periodontal”[All Fields] AND “diseases”[All Fields]) OR “periodontal diseases”[All Fields] OR “periodontitis”[All Fields]) AND (“cardiovascular diseases”[MeSH Terms] OR (“cardiovascular”[All Fields] AND “diseases”[All Fields]) OR “cardiovascular diseases”[All Fields] OR (“cardiovascular”[All Fields] AND “disease”[All Fields]) OR “cardiovascular disease”[All Fields])) AND ((“dimethylarginines”[MeSH Terms] OR “dimethylarginines”[All Fields] OR “asymmetric dimethylarginine”[All Fields]) OR ADMA[All Fields])) AND ((“endothelium”[MeSH Terms] OR “endothelium”[All Fields] OR “endothelial”[All Fields]) AND (“physiology”[Subheading] OR “physiology”[All Fields] OR “function”[All Fields] OR “physiology”[MeSH Terms] OR “function”[All Fields])). Scopus: TITLE-ABS-KEY ((periodontitis OR “periodontal disease”) AND (“cardiovascular disease” OR cvd) AND (Adma OR “asymmetric dimethylarginine”) AND (“endothelial function” OR “endothelial dysfunction”)). Web of Science: TS = ((periodontitis OR “periodontal disease”) AND (“cardiovascular disease” OR cvd) AND (Adma OR “asymmetric dimethylarginine”) AND (“endothelial function” OR “endothelial dysfunction”)). The search was limited to articles published in English, with no date restrictions applied. No filters were used for the study design to ensure a comprehensive search of the available literature.

The inclusion criteria for this systematic review were as follows:

1. Original research articles published in English;
2. Studies investigating the relationship between ADMA, periodontitis, and/or cardiovascular disease;
3. Studies reporting ADMA levels in patients with periodontitis and/or cardiovascular disease;
4. Studies assessing the impact of periodontal treatment on ADMA levels or endothelial function.

The exclusion criteria included the following:

1. Review articles, case reports, editorials, and conference abstracts;
2. Animal or in vitro studies;
3. Studies not reporting ADMA levels or endothelial function outcomes.

To guide our systematic review and ensure a focused approach to addressing our research objectives, we developed a structured PICO (Population, Intervention/Exposure, Comparison, Outcome) framework. This framework, detailed in Table 1, informed our literature search strategy and the selection of relevant studies.

This PICO framework was instrumental in defining our search terms, the inclusion criteria, and the overall scope of our systematic review.

### 2.2. Study Selection and Data Extraction

The initial screening of article titles and abstracts was conducted independently by two team members (B.R and E.F). Articles that passed this initial screening were then subjected to a full-text review to determine their eligibility based on our predetermined criteria. In cases where the two reviewers disagreed, they discussed the issue to reach a consensus. If necessary, a third team member (F.I.) was consulted to resolve any persistent disagreements.

The data extraction process was also carried out independently by two reviewers (B.R. and E.F.) using a custom-designed data collection form. This form was used to systematically record key information from each study, including the following :

Bibliographic details (author, publication year);

Methodological aspects (study design, sample size);

Participant demographics (age, gender);

Clinical characteristics (periodontal and cardiovascular disease status);

Details of interventions (where applicable);

ADMA measurements;

Endothelial function outcomes;

Any other findings relevant to our research question.

In cases where the published information was unclear or incomplete, we reached out to the corresponding authors of the included studies to request additional details or clarifications.

### 2.3. Quality Assessment

To evaluate the methodological rigor of the included studies, we employed the NIH Quality Assessment Tools. Two members of our research team (B.R. and E.F.) independently applied these tools to each study. The NIH tools were selected for their comprehensive approach to assessing various study designs, including observational, case–control, and interventional studies [29].

Our quality assessment process focused on several key aspects:

1. Clarity and specificity of the research question
2. Representativeness of the study sample
3. Reliability and validity of the methods used to measure exposures and outcomes
4. Appropriateness of statistical analyses
5. Consideration and handling of potential confounding variables

Based on these criteria, each study was categorized as “good”, “fair”, or “poor” in terms of overall quality. When the two reviewers’ assessments differed, they engaged in discussion to reach a consensus. In cases where agreement could not be reached, a third reviewer (F.I.) was consulted to make the final determination.

The NIH Quality Assessment Tools were particularly valuable for our review due to their applicability across various study designs, including randomized controlled trials. This allowed for a consistent evaluation approach across our diverse set of included studies [29].

### 2.4. Data Synthesis and Analysis

Due to the heterogeneity in the study designs, populations, and outcome measures, a qualitative synthesis of the included studies was performed. The main findings of each study were summarized and critically evaluated in the context of the research question. Subgroup analyses based on study design, population characteristics, and intervention type were conducted when appropriate. The overall strength of the evidence was evaluated using the Grading of Recommendations, Assessment, Development, and Evaluation (GRADE) approach. The GRADE system considers factors such as study design, risk of bias, consistency, directness, precision, and publication bias to determine the quality of evidence for each outcome. The quality of evidence was categorized as high, moderate, low, or very low. The included studies comprised cross-sectional studies, prospective cohort studies, pilot cohort studies, and randomized controlled trials. This heterogeneity in study design made it challenging to combine the results in a meta-analysis. The included studies have enrolled various patient populations, including patients with periodontitis, cardiovascular diseases, chronic kidney diseases, and a combination of these conditions. This heterogeneity in the population could influence ADMA levels and responses to periodontal treatment. Studies have assessed different outcomes, including serum and salivary ADMA levels, endothelial function, inflammatory markers, and clinical periodontal parameters. This heterogeneity in outcome measures made it difficult to combine the results into a single effect estimate. Not all included studies reported sufficient quantitative data to calculate a pooled effect estimate, such as means and standard deviations of ADMA levels before and after periodontal treatment. Considering these factors, conducting a meta-analysis with the studies included in this systematic review was deemed not inappropriate. Therefore, a qualitative synthesis was carried out.

## 3. Results

**Figure 1:**
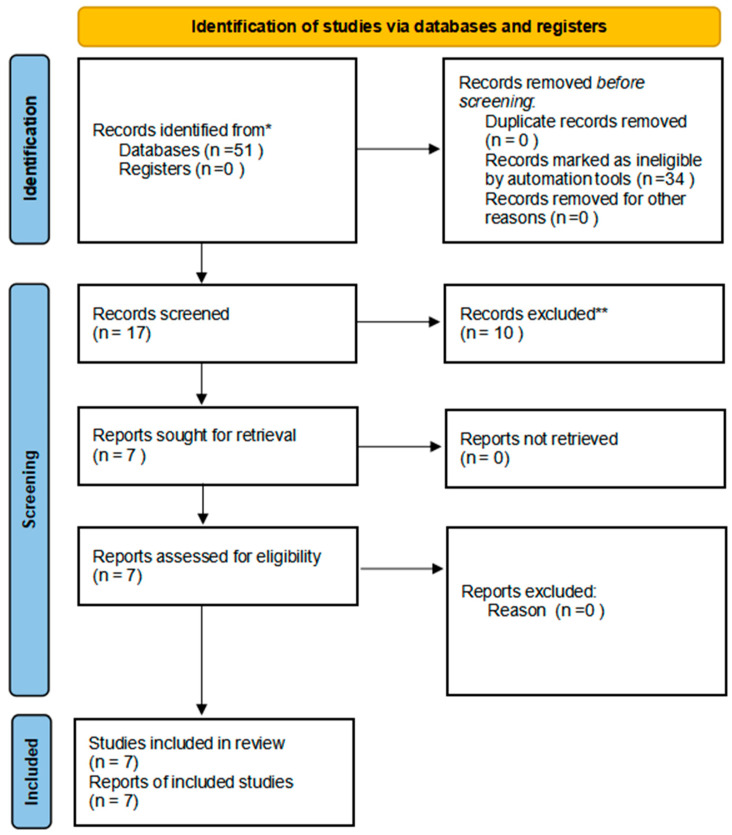
PRISMA flow chart of the included studies. Note. * PubMed, Scopus, and Web of Science; ** Articles do not focus on ADMA.

The initial database search yielded a total of 51 articles: 18 from PubMed, 22 from Scopus, and 11 from Web of Science. After removing duplicates, 34 articles remained for title and abstract screening. Following this screening, 17 articles were selected for full-text assessment. Ten articles were subsequently excluded due to the non-reporting of ADMA levels, a lack of patients with periodontitis or CVD, or being conference abstracts or review articles. Ultimately, seven studies met the inclusion criteria and were incorporated into the qualitative synthesis. The study selection process is illustrated in the PRISMA flow diagram (Figure 1).

The seven included studies comprised three cross-sectional studies, one prospective cohort study, one pilot cohort study, and two randomized controlled trials. These studies collectively involved 1128 participants, with individual study sample sizes ranging from 26 to 392, respectively. Five studies assessed ADMA levels in serum, while two evaluated both serum and salivary ADMA levels. All three cross-sectional studies consistently demonstrated significantly elevated ADMA levels in patients with periodontitis and CVD compared to healthy controls. Two of these studies also reported positive correlations between ADMA levels and clinical periodontal parameters, including probing depth and clinical attachment loss. The prospective cohort study and pilot cohort study examined the effects of periodontal treatment on ADMA levels and endothelial function. Both studies observed significant reductions in ADMA levels following periodontal therapy, with the prospective cohort study additionally reporting improvements in endothelial function. In contrast, the two randomized controlled trials failed to demonstrate significant changes in ADMA levels or endothelial function after periodontal treatment in patients with periodontitis alone. Table 2 presents a concise summary of the key characteristics and findings of the included studies. The studies are categorized as follows: three cross-sectional studies [29,30,31], one prospective cohort study [32], two randomized controlled trials [33,34], and one pilot cohort study [35].

### Quality Assessment Results

The quality assessment of the included studies using the NIH Quality Assessment Tools identified a combination of good- and fair-quality studies (Table 3). Three cross-sectional studies [29,30,31] and one prospective cohort study [32] were rated as being of good quality. One randomized controlled trial [33] was also rated as being of good quality, while another [34] was rated as having fair quality. The pilot cohort study [35] was rated as having fair quality.

The studies rated as being of good quality generally had clear research questions, well-defined study populations, appropriate exposure and outcome measures, and adequate statistical analyses. These studies provided more reliable evidence regarding the relationship between ADMA, periodontitis, and cardiovascular disease, as well as the potential effects of periodontal treatment on ADMA levels and endothelial function. The studies rated as fair quality had some limitations, such as potential selection bias, lack of blinding, or insufficient control for confounding factors.

## 4. Discussion

Our comprehensive analysis integrated findings from a total of seven research papers that explored the interconnections among asymmetric dimethylarginine (ADMA), periodontal disease, and cardiovascular disorders (CVDs). The included studies provided evidence linking periodontitis with endothelial dysfunction and cardiovascular disease risk, potentially mediated by oxidative stress and inflammatory pathways. The relationship between periodontitis, ADMA levels, and CVD risk likely involves complex interactions of inflammatory pathways, oxidative stress, and endothelial dysfunction. Periodontal pathogens and their byproducts may directly or indirectly influence ADMA production and degradation, potentially contributing to systemic vascular effects. Elevated ADMA levels, resulting from impaired dimethylarginine dimethylaminohydrolase (DDAH) activity, inhibit endothelial nitric oxide synthase (eNOS), reducing nitric oxide (NO) production. This mechanism may contribute to endothelial dysfunction in both periodontitis and CVD. While this systematic review has focused primarily on the indirect correlation between periodontitis and cardiovascular disease through ADMA and endothelial dysfunction, it is important to acknowledge that there is substantial evidence for a direct correlation between these two pathologies. This direct link has been extensively studied and provides valuable insights into the complex relationship between oral and cardiovascular health. A notable example of research demonstrating this direct correlation is the work conducted by Pardo et al. [36]. In their study, the researchers found evidence of periodontal pathogens in both oral samples and cardiac specimens of patients undergoing aortic valve replacement. This finding suggests a direct pathway by which oral bacteria associated with periodontitis can translocate to cardiac tissues, potentially contributing to cardiovascular pathology. The presence of oral pathogens in cardiac tissues provides strong support for the concept of direct bacterial invasion as a mechanism linking periodontitis and cardiovascular disease. This direct correlation complements the indirect pathways mediated by inflammatory markers and endothelial dysfunction that we have been discussed in this review. The combination of both direct and indirect pathways underscores the complex and multifaceted nature of the relationship between periodontal and cardiovascular health. 

TAl-Abdulla et al. [33] conducted a prospective cohort study that demonstrated a significant association between successful endodontic intervention and improvements in both metabolic syndrome parameters and systemic inflammatory markers linked to cardiovascular risk. Notably, the study observed reductions in asymmetric dimethylarginine (ADMA), high-sensitivity C-reactive protein (hs-CRP), and matrix metalloproteinase-2 (MMP-2) levels. These findings underscore the potential systemic health benefits of oral interventions, suggesting that the management of chronic apical periodontitis through endodontic procedures may favorably modulate cardiovascular risk factors. The investigation, which employed a two-year follow-up protocol, focused on patients who had undergone either non-surgical root canal retreatment (Re-RCT) or periapical surgery (PS). The researchers assessed multiple parameters, including periapical health status, blood pressure, glycated hemoglobin (HbA1C), lipid profiles, and serum concentrations of inflammatory biomarkers. The recall rate at 2 years was 56.9%, with a 100% radiographic success rate. Serum levels of hs-CRP, ADMA, and MMP-2, as well as HbA1C and lipid levels, were significantly reduced at 2 years compared to pre-operative levels. The pilot cohort study by Almeida et al. [35] demonstrated that periodontal treatment significantly improved clinical parameters and reduced serum ADMA levels in patients with chronic kidney disease and severe periodontitis. This finding suggests that periodontal treatment may have beneficial effects on systemic health by reducing inflammation and improving endothelial function, particularly in patients with severe periodontitis and comorbidities such as chronic kidney disease. The study, conducted on 26 pre-dialysis chronic kidney disease (CKD) patients with severe chronic periodontitis, assessed periodontal parameters, estimated glomerular filtration rate (eGFR), metabolic markers, and ADMA at baseline, 90, and 180 days after periodontal therapy. Periodontal parameters significantly improved 180 days after treatment. Median eGFR significantly increased from baseline to 90 and 180 days, and ADMA levels significantly reduced at 180 days. The results suggest a link between kidney disease, endothelial dysfunction, and periodontitis, and periodontal treatment may benefit CKD course. However, the randomized controlled trials by Okada et al. [34] and Rapone et al. [33] did not find statistically significant differences in ADMA concentration after intensive periodontal treatment, although FMD increased for the test group in Rapone’s study. Okada et al. [34] conducted a parallel group, 3-month follow-up, open-label, randomized controlled trial on 110 patients with early-stage periodontal disease. The control group received standard care, while the test group additionally used a disinfectant mouthwash. Flow-mediated dilation (FMD) and ADMA were evaluated at baseline and at 3 months. The study found no significant improvements in FMD or ADMA in either group at 3 months, despite significant improvements in periodontal parameters in both groups. Advanced periodontal self-care for 3 months did not significantly improve FMD compared to standard care, despite improving periodontal status. Rapone and colleagues [33] conducted a prospective, randomized controlled trial with outcome assessor blinding, encompassing a six-month follow-up period. The study cohort comprised 140 subjects diagnosed with severe periodontitis, specifically selected for the absence of cardiovascular disease (CVD) and traditional CVD risk factors. Participants were randomized in a 1:1 ratio to receive either intensive periodontal therapy (intervention group, *n* = 70) or standard community-based periodontal care (control group, *n* = 70).

The researchers implemented a comprehensive evaluation protocol at three time points: baseline, three months, and six months post-intervention. This protocol included a thorough medical examination, detailed periodontal clinical assessments, quantitative analysis of serum asymmetric dimethylarginine (ADMA) concentrations, and ultrasonographic measurement of flow-mediated dilation (FMD).

Statistical analysis of the data revealed no significant alterations in ADMA concentrations within the intervention group across the study timeline. Moreover, no statistically significant differences in ADMA levels were observed between the intervention and control groups at baseline, or at any subsequent follow-up time points. These findings suggest that intensive periodontal therapy did not significantly impact serum ADMA levels in this cohort of periodontitis patients without pre-existing cardiovascular complications.

However, periodontal parameters decreased after intensive periodontal treatment in the test group, and FMD increased for the test group after therapy. The absence of statistically significant alterations in asymmetric dimethylarginine (ADMA) concentrations observed in these investigations may be attributed to two primary factors: the deliberate exclusion of subjects with cardiovascular risk factors and the relatively brief duration of post-intervention monitoring. In a cross-sectional investigation, Şengül and colleagues [29] conducted a comparative analysis involving 21 individuals diagnosed with advanced generalized periodontitis (classified as Stage III Grade B according to current nomenclature) and 24 subjects with clinically healthy periodontal tissues. Their methodological approach encompassed a comprehensive evaluation of clinical periodontal indices and quantitative analysis of both salivary and serum biomarkers. Specifically, they assessed levels of interleukin-6 (IL-6), nitric oxide synthase (NOS), asymmetric dimethylarginine (ADMA), symmetric dimethylarginine (SDMA), homoarginine (homoArg), arginine, and N-monomethyl-L-arginine (L-NMMA).

The investigators reported statistically significant differences between the periodontitis and control groups across several parameters. Subjects with periodontitis exhibited markedly elevated clinical periodontal indices and salivary concentrations of nitric oxide synthase (NOS), asymmetric dimethylarginine (ADMA), and arginine, with all comparisons yielding *p*-values below 0.05.

Interestingly, the serum analysis revealed a more complex picture. While interleukin-6 (IL-6) levels were significantly higher in the periodontitis group (*p* < 0.05), symmetric dimethylarginine (SDMA) concentrations were notably lower compared to the control subjects (*p* < 0.05).

Furthermore, the researchers identified significant positive correlations between all assessed clinical periodontal parameters and the levels of ADMA, NOS, and arginine (*p* < 0.05 for all correlations). This finding suggests a potential mechanistic link between the severity of periodontal disease and alterations in these biochemical markers. Isola et al. [31] conducted a cross-sectional study including 35 patients with chronic periodontitis (CP), 33 patients with coronary heart disease (CHD), 35 patients with both CP and CHD, and 35 healthy controls. The study assessed clinical and periodontal characteristics, serum, and saliva samples. Patients with CHD and CP+CHD had significantly higher salivary and serum ADMA levels compared to healthy subjects and CP patients. High-sensitivity C-reactive protein (hs-CRP) was a significant predictor of increased salivary and serum ADMA levels. Ferlazzo et al. [30] conducted a cross-sectional study evaluating plasma levels of CoQ10, nitrotyrosine (NT), ADMA, and mRNA levels of inflammatory genes in peripheral blood mononuclear cells (PBMCs) in patients with periodontitis (PT), coronary heart disease (CHD), or both (PT+CHD) compared to healthy controls. PT+CHD patients had significantly lower CoQ10 (*p* = 0.008), higher NT (*p* = 0.005), and higher ADMA levels (*p* < 0.001) compared to controls. Inflammatory gene expression (CASP1, NLRP3, TNF-α) was upregulated in PT, CHD, and PT+CHD groups. The included studies provided evidence linking periodontitis with endothelial dysfunction and cardiovascular disease risk, potentially mediated by oxidative stress and inflammatory pathways. Markers like ADMA, NT, and CoQ10 were found to be altered in patients with both periodontitis and cardiovascular or kidney disease. This systematic review highlights the potential role of ADMA as a biomarker linking periodontitis and CVD. While observational studies consistently show elevated ADMA levels in patients with periodontitis and CVD, interventional studies yield mixed results. This discrepancy in findings between observational and interventional studies highlights the need for further research to elucidate the complex relationship between periodontal treatment, ADMA levels, and cardiovascular outcomes. Several factors may contribute to these mixed results, including differences in study populations, periodontal treatment protocols, follow-up periods, and the presence of comorbidities. Further research is needed to elucidate the precise mechanisms underlying these relationships and to determine the clinical utility of ADMA as a biomarker for assessing cardiovascular risk in patients with periodontitis.

### Limitations

The cross-sectional nature of most studies precludes the establishment of causal relationships, while small sample sizes and potential selection bias due to non-randomization may limit the generalizability of the findings. The heterogeneity in study designs, populations, and outcome measures also limits the comparability of the results and precluded a formal meta-analysis. Despite these limitations, the findings of this systematic review provide valuable insights into the potential role of ADMA as a biomarker for periodontal disease progression and its association with endothelial dysfunction. The heterogeneity in study designs, populations, and outcome measures may also limit the comparability of the results. Despite these limitations, the findings of this systematic review provide valuable insights into the potential role of ADMA as a biomarker for periodontal disease progression and its association with endothelial dysfunction. The regulation of ADMA levels by DDAH emerges as a critical factor in maintaining endothelial function, and impaired DDAH activity may contribute to the pathogenesis of endothelial dysfunction in various disease states. Targeting the ADMA-DDAH pathway could represent a promising therapeutic strategy for the prevention and treatment of endothelial dysfunction and its associated cardiovascular complications. Further research is needed to better understand the influence of periodontitis on ADMA concentrations and endothelial function, particularly in larger study populations and with longer follow-up periods. Additionally, studies investigating the mechanisms underlying the relationship between periodontal health and endothelial function may help to elucidate the potential role of periodontal treatment in reducing cardiovascular risk. Further research is necessary to elucidate the precise mechanisms underlying these relationships and to determine the clinical utility of ADMA as a biomarker for assessing cardiovascular risk in patients with periodontitis. 

## 5. Conclusions

This systematic review provides evidence that ADMA levels are elevated in patients with periodontitis and CVD compared to healthy controls. Periodontal treatment may reduce ADMA levels and improve endothelial function, particularly in patients with comorbidities, although results are not uniform across all studies. These findings suggest that ADMA could be a promising biomarker linking periodontitis, endothelial dysfunction, and CVD. However, further high-quality longitudinal studies are needed to confirm its clinical utility and to elucidate the mechanisms underlying these relationships. The integration of periodontal care into CVD prevention and management strategies warrants consideration, as it may contribute to reducing the cardiovascular risk associated with periodontitis, but additional research is necessary to confirm this approach. ADMA, an endogenous inhibitor of NOS, can lead to endothelial dysfunction when elevated. The chronic inflammatory state in periodontitis may contribute to increased ADMA levels, providing a potential mechanistic link between periodontitis and cardiovascular disease. Further research is needed to fully elucidate the role of ADMA in this relationship and to explore the potential of ADMA as a biomarker and therapeutic target.

## Figures and Tables

**Table 1:** PICO framework for the systematic review.

Component	Description
Population	Adult patients with periodontitis and/or cardiovascular diseases
Intervention/Exposure	Presence of periodontitis or periodontal treatment
Comparison	Absence of periodontitis or no periodontal treatment
Outcome	ADMA levels and/or endothelial function

**Table 2:** Summary of included studies.

Study	Study Design	Population	Methods	Key Findings
Şengül et al. [29]	Cross-sectional	The study included two groups: individuals diagnosed with advanced generalized periodontitis (Stage III Grade B, according to the current classification) and a control group of periodontally healthy subjects.	The researchers assessed various periodontal clinical indicators and conducted analyses of both saliva and blood samples. The analysis examined a range of compounds crucial for inflammation, endothelial function, and nitric oxide production. Among these, arginine and its methylated derivatives—asymmetric dimethylarginine (ADMA), symmetric dimethylarginine (SDMA), N-monomethyl-L-arginine (L-NMMA), and homoarginine (homoArg)—play key roles in vascular regulation. Additionally, nitric oxide synthase (NOS), the enzyme responsible for nitric oxide synthesis, and interleukin-6 (IL-6), an important inflammatory mediator, were evaluated.	The analysis revealed that patients with periodontitis exhibited significantly elevated concentrations of nitric oxide synthase (NOS), asymmetric dimethylarginine (ADMA), and arginine in their saliva compared to the control group.
Ferlazzo et al. [30]	Cross-sectional	The study encompassed four distinct groups: individuals diagnosed with periodontitis (PT), subjects with confirmed coronary heart disease (CHD), patients presenting with both conditions simultaneously (PT+CHD), and a cohort of healthy participants serving as controls.	A comprehensive analysis of blood samples, focusing on several key components: plasma concentrations of coenzyme Q10 (CoQ10), nitrotyrosine (NT), and asymmetric dimethylarginine (ADMA). Additionally, the researchers examined the expression levels of genes associated with inflammation in peripheral blood mononuclear cells (PBMCs) by quantifying their messenger RNA (mRNA).	PT+CHD patients had significantly lower CoQ10, higher NT, and higher ADMA levels compared to controls; inflammatory gene expression upregulated in PT, CHD, and PT+CHD groups.
Isola et al. [31]	Cross-sectional	Individuals diagnosed with chronic periodontitis (CP), subjects with confirmed coronary heart disease (CHD), patients presenting with both conditions concurrently (CP+CHD), and a cohort of healthy participants serving as controls.	The study protocol involved a comprehensive assessment of clinical and periodontal characteristics. Additionally, biological samples, including both serum and saliva, were collected from all participants. These samples were subsequently analyzed to quantify the levels of asymmetric dimethylarginine (ADMA) and high-sensitivity C-reactive protein (hs-CRP).	The analysis revealed that subjects with coronary heart disease, both with and without concurrent chronic periodontitis, exhibited significantly elevated concentrations of ADMA in both salivary and serum samples. The concentrations detected in these groups significantly exceeded those found in both the healthy control subjects and individuals diagnosed solely with chronic periodontitis.
Al-Abdulla et al. [32]	Prospective cohort (2-year follow-up)	Patients who had received one of two types of endodontic interventions: either a non-surgical revision of previous root canal therapy or a surgical procedure addressing periapical lesions.	A comprehensive assessment included multiple parameters: the condition of periapical tissues, measured systemic blood pressure, and analyzed blood samples for glycated hemoglobin (HbA1c) and lipid profiles. Additionally, the researchers quantified the serum levels of specific inflammatory markers, including high-sensitivity C-reactive protein (hs-CRP), asymmetric dimethylarginine (ADMA), and matrix metalloproteinase-2 (MMP-2).	Serum levels of hs-CRP, ADMA, MMP-2, HbA1C, and lipid levels were significantly reduced at 2 years compared to pre-operative levels. The analysis of blood samples taken two years post-intervention revealed a significant decrease in several key biomarkers compared to their pre-operative values. Specifically, the researchers observed marked reductions in serum concentrations of high-sensitivity C-reactive protein (hs-CRP), asymmetric dimethylarginine (ADMA), and matrix metalloproteinase-2 (MMP-2). Additionally, glycated hemoglobin (HbA1c) levels and various lipid parameters showed substantial improvements over the two-year period.
Rapone et al. [33]	Randomized controlled trial (6-month follow-up)	Individuals diagnosed with advanced periodontal disease who had no history of cardiovascular disease (CVD) or presence of traditional CVD risk factors.	The study protocol included comprehensive evaluations at three time points: before treatment, and at three- and six-months post-intervention. These assessments encompassed a thorough medical examination, detailed periodontal clinical measurements, laboratory quantification of asymmetric dimethylarginine (ADMA) levels, and ultrasonographic evaluation of flow-mediated dilation (FMD).	Following intensive periodontal therapy, the researchers found no statistically significant alterations in ADMA concentrations. However, the group receiving the intervention demonstrated an improvement in flow-mediated dilation, suggesting enhanced endothelial function.
Okada et al. [34]	Randomized controlled trial (3-month follow-up)	Individuals diagnosed with incipient periodontal disease, representing the early stages of the condition.	Assessments of flow-mediated dilation (FMD) and serum asymmetric dimethylarginine (ADMA) concentrations. These measurements were conducted at the initiation of the study and again after a three-month interval.	Despite observed improvements in clinical periodontal parameters, the analysis revealed no statistically significant changes in either flow-mediated dilation or serum ADMA levels in any of the study groups at the three-month follow-up evaluation.
Almeida et al. [35]	Pilot cohort	Individuals diagnosed with advanced chronic periodontitis who were also in the pre-dialysis stage of chronic kidney disease.	The study protocol included a comprehensive assessment of multiple parameters at three distinct time points: before treatment, and at 90 and 180 days following periodontal intervention. The evaluated measures encompassed periodontal clinical indicators, estimated glomerular filtration rate (eGFR), various metabolic markers, and serum asymmetric dimethylarginine (ADMA) concentrations.	Six months following periodontal therapy, the analysis revealed significant positive changes across several parameters. The researchers observed marked improvements in periodontal clinical indicators, a notable increase in estimated glomerular filtration rate, and a substantial reduction in serum ADMA levels.

Note. ADMA, asymmetric dimethylarginine; CHD, coronary heart disease; CKD, chronic kidney disease; CoQ10, coenzyme Q10; CP, chronic periodontitis; CVD, cardiovascular disease; eGFR, estimated glomerular filtration rate; FMD, flow-mediated dilation; hs-CRP, high-sensitivity C-reactive protein; IL-6, interleukin-6; MMP-2, matrix metalloproteinase-2; NOS, nitric oxide synthase; NT, nitrotyrosine; PBMCs, peripheral blood mononuclear cells; PS, peri-apical surgery; PT, periodontitis; Re-RCT, root canal re-treatment; SDMA, symmetric dimethylarginine; TNF-α, tumor necrosis factor-alpha.

**Table 3:** Quality assessment of all included studies using the NIH Quality Assessment Tools.

Study	Study Design	Quality Rating	Risk of Bias
Şengül et al. [29]	Cross-sectional	Good	Low
Ferlazzo et al. [30]	Cross-sectional	Good	Low
Isola et al. [31]	Cross-sectional	Good	Low
Al-Abdulla et al. [32]	Prospective cohort	Good	Low
Rapone et al. [33]	Randomized controlled trial	Good	Low
Okada et al. [34]	Randomized controlled trial	Fair	Moderate
Almeida et al. [35]	Pilot cohort	Fair	Moderate

## Data Availability

Not applicable.
